# Observation of site-selective chemical bond changes via ultrafast chemical shifts

**DOI:** 10.1038/s41467-022-34670-2

**Published:** 2022-11-22

**Authors:** Andre Al-Haddad, Solène Oberli, Jesús González-Vázquez, Maximilian Bucher, Gilles Doumy, Phay Ho, Jacek Krzywinski, Thomas J. Lane, Alberto Lutman, Agostino Marinelli, Timothy J. Maxwell, Stefan Moeller, Stephen T. Pratt, Dipanwita Ray, Ron Shepard, Stephen H. Southworth, Álvaro Vázquez-Mayagoitia, Peter Walter, Linda Young, Antonio Picón, Christoph Bostedt

**Affiliations:** 1 Chemical Sciences and Engineering Division, Argonne National Laboratory, Argonne, IL 60439 USA; 2 Paul-Scherrer Institute, CH-5232 Villigen PSI, Switzerland; 3 Departamento de Química, Universidad Autónoma de Madrid, 28049 Madrid, Spain; 4 LUXS Laboratory for Ultrafast X-ray Sciences, Institute of Chemical Sciences and Engineering, École Polytechnique Fédérale de Lausanne (EPFL), CH-1015 Lausanne, Switzerland; 5 Laboratory of Theoretical Physical Chemistry, Institute of Chemical Sciences and Engineering, École Polytechnique Fédérale de Lausanne (EPFL), CH-1015 Lausanne, Switzerland; 6 SLAC National Accelerator Laboratory, Menlo Park, CA 94025 USA; 7 Stanford PULSE Institute, SLAC National Accelerator Laboratory, Menlo Park, CA 94025 USA; 8 Argonne Leadership Computing Facility, 9700 S. Cass Avenue, Lemont, IL 60439 USA; 9 Department of Physics and James Franck Institute, The University of Chicago, Chicago, IL USA

**Keywords:** Atomic and molecular interactions with photons, Physical chemistry, Chemical physics

## Abstract

The concomitant motion of electrons and nuclei on the femtosecond time scale marks the fate of chemical and biological processes. Here we demonstrate the ability to initiate and track the ultrafast electron rearrangement and chemical bond breaking site-specifically in real time for the carbon monoxide diatomic molecule. We employ a local resonant x-ray pump at the oxygen atom and probe the chemical shifts of the carbon core-electron binding energy. We observe charge redistribution accompanying core-excitation followed by Auger decay, eventually leading to dissociation and hole trapping at one site of the molecule. The presented technique is general in nature with sensitivity to chemical environment changes including transient electronic excited state dynamics. This work provides a route to investigate energy and charge transport processes in more complex systems by tracking selective chemical bond changes on their natural timescale.

## Abstract (web summary)

X-ray photoelectron spectroscopy probes the chemical environment in a molecule at a specific atomic site. Here the authors extend this concept with a site selective trigger to follow chemical bond changes as they occur on the femtosecond time scale.

## Introduction

Chemical changes triggered by photoexcitation may occur as fast as a few femtoseconds (10^−15^ s) and involve the complex interplay between electron rearrangement and nuclear motion. The underlying dynamics are commonly investigated with pump and probe approaches, where valence electron photoexcitation can drive molecules impulsively into out-of-equilibrium states and subsequent sampling of the system can reveal a wealth of information about the reaction pathways^1^. Using shorter wavelengths reaching the x-ray regime offers the opportunity to reach higher temporal and atomic spatial resolutions^2,3^. Adding local chemical information to the ever-increasing time resolution is highly desired. This can be achieved through x-ray photoelectron spectroscopy, a prime tool for the investigation of electronic properties of matter^4^. The binding energy of core electrons is sensitive to the specific chemical environment of the ionizing atom, i.e., the surrounding electron density and molecular structure, that can be expressed in a chemical shift. This chemical shift is exploited for a wide variety of applications in chemistry, physics, and material science. So far, XPS studies are mostly restricted to the static domain. However, strong chemical changes may be expected when the system is driven out of equilibrium, even when those are induced by electron rearrangements before the onset of nuclear motion. Recent studies^5,6^ have partially shown the potential of this technique to follow UV-excited state dynamics, but a higher temporal resolution was desired in order to track the transient evolving system via chemical shifts right after excitation^7^.

In this work we apply the concept of chemical shifts to the few femtosecond regime and extend it with a site-selective trigger. We approach the natural timescale of electron motion and show the sensitivity of chemical shifts to excited electronic state dynamics, electron rearrangement and the correlated nuclear motion. In this combined experimental and theoretical approach, we detect ultrafast changes in the chemical environment triggered by a site-selective initial excitation in an hetero-site x-ray pump/x-ray probe scheme. In particular, we observe the XPS signature of core-excited states and of the dissociation during and after Auger decay. Our approach is complementary to recent advances in x-ray pump/probe absorption spectroscopy probing the unoccupied states^8–12^. The real-time observation of the chemical environment changes around a specific atom right after photoexcitation, during and after Auger processes sheds light on the most fundamental dynamics taking place in molecules^13^. This concept can be readily extended to site-specific charge migration dynamics in more complex systems and into the attosecond domain^3,14,15^.

## Results

**Fig. 1:**
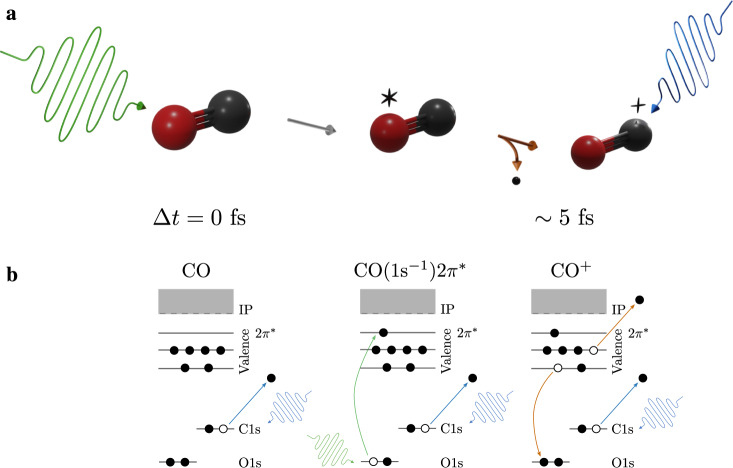
Site-selective two-color femtosecond x-ray pump-probe scheme to follow ultrafast chemical shifts in CO. **a** The x-ray pump (green) excites the O 1*s* electron, leading to the formation of CO(1*s*^−1^)2π^*^, which subsequently decays in few femtoseconds via Auger processes (orange) to CO^+^. A time-delayed x-ray pulse (blue) probes the dynamics by ionizing a C1*s* electron. **b** Scheme of the energy levels of the probed states at each time delay: ground state, core-excited state and Auger states.

The concept of the ultrafast and site selective resonant pump, XPS probe experiment is summarized in Fig. 1a. The x-ray pump pulse promotes a core electron from the O atom to an unoccupied 2π^*^ orbital, leading to the formation of a neutral bound core-excited state CO(1*s*^−1^)2π^*^, as depicted in Fig. 1b. This unstable state decays in a few femtoseconds via Auger processes, leading to the ejection of an Auger electron and the formation of multiple dissociative and bound cationic states CO^+^, characterized by a hole in the valence shell. The time-delayed x-ray probe pulse ionizes a 1*s* electron from the C atom. By measuring the photoelectron kinetic energies, we obtain the corresponding binding energies and associated chemical shifts. The measured XPS spectra are processed and normalized. The spectral and temporal resolutions are defined by the FEL parameters and are estimated to 3.5 eV and ≤10 fs, respectively^16^. The time delay between the two pulses was scanned between −5 and 40 fs. To describe the correlated electronic and nuclear dynamics, we developed an accurate model that treats both electron and nuclear motions at the quantum level. A detailed description of the theoretical model and experimental set-up is given in Methods section and Supplementary Materials.

**Fig. 2:**
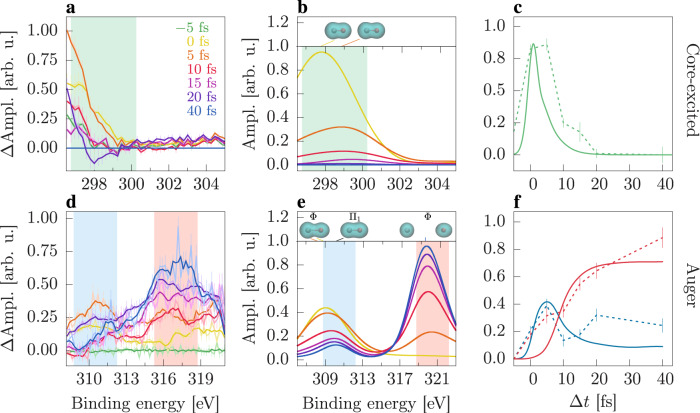
Time-dependent XPS spectra and transient signals of core-excited and Auger states. Panels **a** and **d** show the measured time-resolved XPS spectra. The corresponding calculated XPS spectra are displayed in panels **b** and **e**. The experimental XPS spectrum of the core-excited state shown in panel **a** is obtained by subtracting the signal at 40 fs and ΔAmpl. denotes the resulting difference signal. In panel **d** ΔAmpl. indicates the spectral amplitude after subtraction of the negative time delay signal. The initial time coincides with the center of the pump pulse. In the insets of panels **b** and **e**, the electron density in the core-excited and the dominant dissociative Φ and bound Π Auger states are displayed. The transient signals as a function of the time delay, panels **c** and **f**, are obtained by integrating the spectra over an energy window of 3.5 eV centered at the relevant peaks (shaded green, blue and red areas). The experimental and theoretical signals are displayed as dashed and solid lines, respectively. The vertical lines depict the standard error of the data as discussed in Supplementary Material S1.0.6.

The measured and calculated time-resolved C1*s* XPS spectra of the core-excited state are shown in Fig. 2a and b, respectively. The core-excited state consists of an highly excited but still neutral molecule. This state is unstable with a 4.2 fs lifetime^17^, and it decays primarily through Auger processes. For the core-to-valence excitation, we calculate an associated chemical shift of ~2.4 eV above the ground state. In the experimental data, the core-excited state contribution to the spectra is overlaid with the dominant ground state photoline at ~298 eV and appears as a transient tail of the XPS spectrum of the ground state due to the bandwidth of the pulses (c.f. Supplementary Fig. S2 in the Supplementary Materials). In the difference signal plotted in Fig. 2a, the core-excited state signal can be clearly identified. The data analysis routines are explained in detail in Supplementary Section S1.A of the Supplementary Materials. We note that the calculated spectra yield more details in the chemical shift beyond the energy resolution of the current experiment, capturing also the electronic rearrangement and onset of nuclear motion (refer to inset in Fig. 2b) on the few-femtosecond time scale as we will discuss below. Figure 2c shows a comparison between the experimental and calculated transient behavior of the core-excited state signature. The transients are obtained by integrating the XPS spectra in a 3.5 eV energy window (colored regions). The signal in both calculated and measured transients promptly appears at 0 fs and decays rapidly in less than 10 fs. The observed transient agrees with the short lifetime of the core-excited state, and the fast depletion into the Auger states. The experimental data are slightly temporally broader due to limitation in fully correcting timing jitter and accounting for fluctuating pulse structure of the SASE pulses (c.f. Supplementary Section S1.A.4 in Supplementary Materials). Despite these experimental limitations, the experimental results capture the fast rise and decay of the core excited states in agreement with the prediction.

The decay of the core-excited state is accompanied by a simultaneous rise of an Auger signal. The experimental and theoretical time-resolved C1*s* XPS spectra of CO^+^ are presented in Fig. 2d and e, respectively. The cationic Auger states exhibit a strong increase of the binding energy compared to the neutral molecule. As a multitude of Auger states are populated^18^, the corresponding XPS spectra cover a wide energy range from 305 eV up to 325 eV. The spectra present analogous features with two peaks at ~310 eV and ~317 eV (theory ~320 eV). The theoretical spectrum slightly overestimates the chemical shifts by ~3 eV. The calculated error for the excited Auger states is slightly worse than state-of-the art calculations for the core-ionization potential in neutral CO with <1 eV^19^ but within the expected range of 1% for excited-state calculations^20^. Such a level of discrepancy can be expected due to the complexity of describing strong electron correlation in core-hole states. In particular, we find that at long internuclear distances, (more than 50) highly excited core-hole (satellite) states are populated, see Supplementary Fig. S6 in Supplementary Materials. We can provide an accurate description of the strong electron correlation for the 10 lowest-excited states by using state-average calculations at CASSCF level. However, this approach is not computationally feasible for the highly excited satellite states, which we treat therefore at the configuration interaction level without further relaxation of the orbitals. A more exhaustive discussion is provided in Supplementary Section S1.B.4 of the Supplementary Materials.

In the following we discuss the different temporal behaviors of the two main features, which are related to the dissociative or bound Auger states. Similar to the discussion of the core-excited state, we integrate the spectral intensity in the blue and red shaded area of the Auger spectra and display the temporal evolution in panel 2f. The low binding energy peak exhibits a delayed rise compared to the core-excited state with its peak intensity at ~5 fs, followed by a persistent signal decay. The decay is not as pronounced in the experimental data in panel 2d which are limited by the statistics and challenges in proper background subtraction (refer to Supplementary Section S1.A.6 in Supplementary Materials). Our calculations show that this peak includes the contribution of all Auger – both dissociative and bound – states, where the bound states are responsible for the residual signal with long lifetime (>40 fs). The higher energy peak at ~317 eV (theory ~320 eV) exhibits a further delayed onset (~5 fs) in the calculated data, while the measured signal already shows some spectral intensity at <5 fs delay. Both data sets show persistent high spectral intensity for the long time delay which saturates at ~40 fs. This feature is attributed to the dissociative Auger states increasing in population concomitant with the decay of both, the core-excited state and the Auger feature at ~310 eV while the molecule fragments. This double peak structure changing in time has also been observed in previous calculations for CH_3_I dissociation^7^. Despite the mentioned limitations of the experimental data in terms of statistics and background subtraction, the measured and simulated transient signals shown in Fig. 2f are in good agreement, demonstrating population transfer of the core-excited state to the Auger decay channels on the few-femtosecond time scale.

## Discussion

**Fig. 3:**
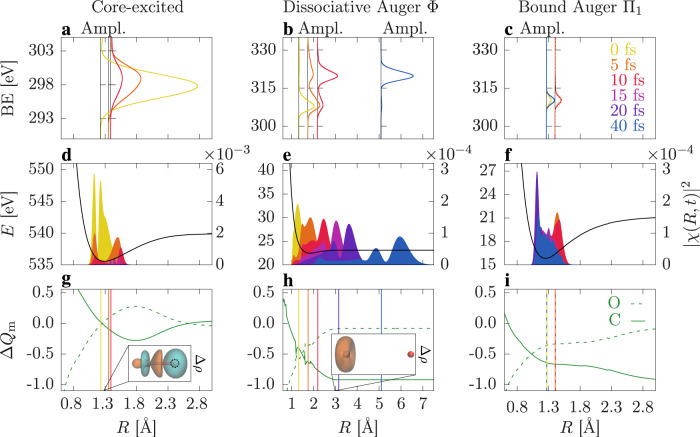
Impact of the electron density and nuclear dynamics on the chemical shift. Calculated time-resolved C1*s* XPS spectra of the (**a**) core-excited state, (**b**) dissociative Φ Auger state, and (**c**) bound Π_1_ Auger state, for different time delays. The spectra are displayed at the average position of the nuclear wavepacket at the respective time. The spectra are color coded by the time delay displayed in panel (**c**). Panels **d**–**f** show the corresponding potential energy curves together with the nuclear wavepacket at different time delays. The difference in Mulliken charges Δ*Q*_m_ at the C (solid line) and O (dashed line) sites, between the corresponding state and the ground state are shown in panels **g**–**i**. The average position of the nuclear wavepacket at different time delays is indicated as vertical bars. The electron density difference (Δ*ρ*) between the excited states and the ground state is given in the insets, where blue and orange densities correspond to gain and loss with respect to the ground state, respectively. The core vacancy at the O site is highlighted by the dashed circle.

In the next step, we analyze and correlate the time-dependent chemical shifts to the ultrafast changes in electron density and nuclear motion in our theoretical framework. The calculated time-resolved XPS spectra associated with the core-excited, the dominant dissociative, and bound Auger states are presented in Fig. 3a, b and c, respectively. The spectra are displayed at the average position of the nuclear wavepacket at the corresponding time. The evolving nuclear wavepackets together with the potential energy curves are depicted in Fig. 3d–f. We also analyze the local charge at each atomic center, the so-called Mulliken charges *Q*_m_^21^, following the ultrafast dynamics (c.f. Supplementary Section S2.A for further discussion). We show the differences in Mulliken charges Δ*Q*_m_ between each state and the ones in the ground state in its equilibrium position (*R* ~ 1.14 Å) in Fig. 3g, h and i. Similarly, the total Mulliken charges are presented in Supplementary Fig. S7 in Supplementary Materials. We first consider the hole dynamics during the formation and decay of the core-excited state. The promotion of a localized O1*s* electron to a 2π^*^ orbital induces changes in the electron density in order to screen the core hole created at the oxygen site. These effects produce an elongation of the bond length by ~0.14 Å in the bound core-excited state as shown in Fig. 3d, and a withdrawal of electron density from the chemical bond towards the O site depicted Fig. 3g.

A chemical shift of 2.4 eV with respect to the ground state signal is calculated, which is purely driven by photoexcitation and electron screening of the core vacancy. The chemical shift further increases due to the bond stretching in the short-lived transient state. Upon a closer look we note that the theory predicts a consistent shift as a function of delay that is not visible in the data in Fig. 2a. The data shows within the experimental uncertainties mostly a change in intensity of the signal. We attribute this difference between theory and experiment to the modeling of the excitation. In the theoretical description we assume a transform-limited pulse that creates a well-defined wavepacket. The evolution of the coherent wavepacket is presented in Fig. 3d, showing a bimodal distribution, which arises from interferences during the photoexcitation, with a more populated peak at longer internuclear distances. Further information regarding the impact of the pulse duration on the wavepacket distribution can be found in the Supplementary Material (c.f. Supplementary Section S2.B). The experiment utilizes self-amplified spontaneous emission (SASE) pulses instead, whose temporal and energy structure changes shot by shot. Thus in the experimental data, on average, a nuclear wavepacket distribution is expected around the mean internuclear distance of ~1.28 Å with a related chemical shift of 2.4 eV that does not significantly change with the time delay. In conjunction to the chemical shifts, the total signal intensity of the core-excited state vanishes on the same time-scale as the core-hole decay of 4.2 fs, yielding almost no core-excited state signature in the XPS spectra after 15 fs time delay. Despite the limited spectral resolution, the experimental data presented in Fig. 2a show a rise and decay of the signal as a shoulder to the ground state XPS.

The time-dependent XPS spectra of the cation Auger states can also be correlated to the transient electron density. Around 86% of the channels lead to dissociation and the rest of the states remain bound. For the sake of clarity, we discuss here only the dominant dissociative (Φ) and bound (Π_1_) Auger states, which account for ~17.9 % and ~8.9 % of the total Auger yield, respectively^18^. The same qualitative trend of the chemical shifts in terms of the hole density can be bring for the other states. Note that the calculated time-resolved XPS spectrum shown in Fig. 2e includes all dissociative and bound Auger states, while only the contribution of the dominant bound and dissociative Auger states are displayed in Fig. 3e and f, respectively.

The Φ Auger state is dissociative, i.e., the nuclear wavepacket moves towards larger internuclear distances, presented in Fig. 3e. In the course of molecular fragmentation, the valence hole ends up trapped at the C site, the changes of the electron density Δ*ρ* for a long internuclear distance are depicted in the inset of Fig. 3h. Mulliken charge analysis also reflects how the valence hole is shared between the two atoms in the early times of the dynamics and then moves towards the C site. The rise of local positive charge at the C site leads in turn to an increase of binding energy of the C1*s* electron. This is observed in the time-resolved XPS spectrum of Fig. 3b which arises at ~11 eV higher in energy compared to the one at 0 fs. Both clearly separated features are observed in the experimental data (Fig. 2d) with the same transfer of spectral intensity from the lower to the higher binding energy peak, as shown in Fig. 2f.

A different behavior for bound and quasi-bound Auger states is observed. Several vibrational states are populated in those channels presenting small variations in the bond distance, as illustrated in the nuclear dynamics of the Π_1_ state in Fig. 3f. The time-resolved spectrum does not exhibit any noticeable chemical shift in time and remains centered around 310 eV as shown in Fig. 3c. This is in agreement with experimental spectra and transient signals presented in Fig. 2d, f. The molecular vibration produces slight oscillations in the partial charges at the C and O sites, depicted in Fig. 3i, but the valence hole mainly remains delocalized on both sites and explains the static chemical shifts observed for bound states.

Our study shows that the chemical shift in XPS is sensitive to electronic excitations and correlated nuclear dynamics on the femtosecond time scale. Already purely electronic excitations lead to an appreciable chemical shift that can be followed with femtosecond time-resolution with 500 eV photon energy pulses available from XFELs^22^ and table-top laser sources^23^. Exploiting the chemical shifts from core electrons will lead to ample new opportunities to understand the ultrafast evolution of excited states in molecules and condensed matter. Extending the approach of a site selective x-ray pump to electronic Raman processes^24,25^ or the targeted optical excitation of chromophores^15^ opens the door for resolving charge dynamics in complex systems in space and time. Attosecond x-ray pulses^26^ paired with the next-generation superconducting XFELs will allow us to follow charge migration or more general electron correlations-driven phenomena via chemical shifts.

## Methods

### Experimental setup

The experiment was carried out at the AMO endstation of the Linac Coherent Light Source (LCLS) free electron laser^27^. The “Fresh slice" operation mode was used to generate a pair of collinear time-delayed femtosecond x-ray pulses^16,28^. The time delay between the pump and probe pulses was scanned between −5 and 40 fs at discrete steps and with a jitter of 3 fs. Both pulses were focused to a spot size of nominally ~1–2 μm using a pair of Kirkpatrick–Baez mirrors. An hemispherical electron analyzer (Scienta EW4000) was adapted to the specific requirements for experiments at the LCLS and integrated into the AMO endstation. The sample molecules were delivered with a pulsed valve to the interaction point which allowed the delivery of a high-density gas target (10^18^ molecules/m^3^) at the interaction point, while keeping the pressure in the experimental chamber and spectrometer at 10^−7^ mbars. Down stream of the electron analyzer, a compact ion time-of-flight (iTOF) analyzer was used for absolute energy calibration based on total ionization yield of CO gas, in addition to the diagnostic tools offered by LCLS. Further details on the experimental setup and data processing are given in the Supplementary Materials.

### Data collection and reduction

The data from the accelerator, the lasing diagnostics, and from the experiment were collected on a shot-by-shot basis and tagged with a unique pulse id. The hemispherical electron spectrometer was also outfitted with a charged coupled device (CCD) camera compatible with the LCLS data acquisition system. This allowed the correlation of the lasing process and experimental data on a shot-to-shot basis and accordingly a binning of the data for improved time and spectral resolution. To ensure resonant excitation and energy correction for the XPS spectra, the absolute energy of the individual pulses was characterized. Using information from the XPS spectra of the C1*s* photolines, the iTOF yield, the gas energy monitor (GMD) and the transverse cavity (XTCAV)^29^, information about the photon energy, pulse energy, and time delay could be extracted for each of the pump and probe pulses. After data sorting and correction, the experimental resolution was approximated to 6 fs in time and 3.5 eV in energy. A detailed description of the data sorting and reduction is presented in the Supplementary Materials.

### Quantum model

We developed a quantum model to provide a complete picture of the ultrafast electron and molecular rearrangements following core-excitation of molecules and interpret the processes into play by calculating the time-dependent chemical shifts. The dynamics triggered by the X-ray pump pulse in the ground, the core-excited and the multiple Auger states is described by solving the time-dependent Schrödinger equation (TDSE) at zeroth-order in time-dependent perturbation theory (TDPT) with respect to the x-ray probe pulse, i.e., only the pump pulse considered. The interaction between the pump pulse and the molecule is described by a transition dipole operator evaluated in the length gauge. The Auger transitions are also included in the TDSE and they couple the core-excited state with the different Auger states, accounting also for the Auger electron in the continuum. We solve the TDSE for the nuclei for different time delays between the two pulses from 0 to 40 fs, using discrete grids in time, space (the internuclear distance *R*) and Auger electron energy^30^. The Gaussian laser pulse of 10 fs duration is polarized perpendicular to the molecular axis in the direction of maximum excitation of the O 1*s* → 2π^*^ transition. The Auger decay is represented phenomenologically via Auger decay widths in the equations of motion of the core-excited state, as well as in the Auger states created by the depletion of the transient species. The Auger decay widths are taken from the literature^18^. The photoelectron yield is calculated at first order in TDPT within the rotating wave and short pulse approximations. The yield mainly depends on the population in the state that is ionized by the x-ray probe pulse and the transition dipole moments between this state and all core-hole and satellites states.

The potential energy curves of the relevant electronic states (the ground, core-excited, Auger, main core-hole, and satellite states) of different symmetries and spin are calculated at the configuration interaction (CI) level of theory to access the highly excited states, using molecular orbitals optimized previously in a state-average complete active space self-consistent field (CASSCF) calculation over several low-lying excited states^31,32^. This method allows us to include most of the strong electron relaxation in the presence of a core vacancy. Therefore, our model describes on an equal footing the electron and nuclear dynamics and accounts for all relevant channels open upon x-ray core-excitation of our benchmark CO molecule, and could be extended to the treatment of larger systems. Further details on the model can be found in the supplementary materials.

## Supplementary information

Supplementary Information

Peer Review File

## Data Availability

The processed data used in this study is available in the Zenodo.org database under 10.5281/zenodo.7074867 accession code [10.5281/zenodo.7074867].

## Code availability

The data analysis codes are available upon request. The codes for the theoretical model are available in a collaborative manner upon request.
