# Supplementary material for: Observation of site-selective chemical bond changes via ultrafast chemical shifts
Source: Nat Commun. 2022 Nov 22;13:7170. doi: 10.1038/s41467-022-34670-2 (PMC9684563; doi:10.1038/s41467-022-34670-2)
Supplement: Supplementary file 1 — Supplementary Information [file 41467_2022_34670_MOESM1_ESM.pdf]

Supplementary Materials:
Observation of site-selective chemical bond changes via ultrafast chemical shifts

Andre Al-Haddad,[1,2] Solène Oberli,[3,4,5] Jesús González-Vázquez,[3] Maximilian Bucher,[1] Gilles Doumy,[1] Phay Ho,[1] Jacek Krzywinski,[6] Thomas J. Lane,[6] Alberto Lutman,[6] Agostino Marinelli,[7,6] Timothy J. Maxwell,[6] Stefan Moeller,[6] Stephen T. Pratt,[1] Dipanwita Ray,[6] Ron Shepard,[1] Stephen H. Southworth,[1] Alvaro Vazquez-Mayagoitia,[8] Peter Walter,[6] Linda Young,[1,9] Antonio Picón,[1,3] and Christoph Bostedt[1,2,4]

[1]*Chemical Sciences and Engineering Division, Argonne National Laboratory, Argonne, Illinois 60439, USA*

[2]*Paul-Scherrer Institute, CH-5232 Villigen PSI, Switzerland*

[3]*Departamento de Química, Universidad Autónoma de Madrid, 28049 Madrid, Spain*

[4]*LUXS Laboratory for Ultrafast X-ray Sciences, Institute of Chemical Sciences and Engineering, École Polytechnique Fédérale de Lausanne (EPFL), CH-1015 Lausanne, Switzerland*

[5]*Laboratory of Theoretical Physical Chemistry, Institute of Chemical Sciences and Engineering, École Polytechnique Fédérale de Lausanne (EPFL), CH-1015 Lausanne, Switzerland*

[6]*SLAC National Accelerator Laboratory, Menlo Park, CA 94025, USA*

[7]*Stanford PULSE Institute, SLAC National Accelerator Laboratory, Menlo Park, CA 94025, USA*

[8]*Argonne Leadership Computing Facility, 9700 S. Cass Avenue, Lemont IL 60439, USA*

[9]*Department of Physics and James Franck Institute, The University of Chicago*

S1. SUPPLEMENTARY METHODS

A. Experimental techniques

1. *Hemispherical electron analyzer*

The XPS data were recorded with a Scienta EW4000 hemispherical electron analyzer. The operation mode of the spectrometer was adapted to the specific requirements of single-shot data acquisition at XFEL sources. Each individual XPS spectrum was acquired with XFEL beam rate and tagged with a pulse IDs to correlate it with the other XFEL parameters and data. For that purpose the detector camera imaging the phosphor was replaced with an OPAL 2.0 which is fully integrated into the LCLS data acquisition system and capable of recording images at the XFEL beam rate of 120 Hz. The spectrometer was operated in Fixed Analyzer Transmission mode (FAT), setting a fixed pass energy at 200 eV and changing the central kinetic energy to the desired energy window with (16 eV). A transmission correction curve was experimentally determined in order to obtain the correct spectral intensities. For that purpose we set the spectrometer at a pass energy E_{pass}= 200 eV and a kinetic energy E_{kin}= 234 eV, and subsequently scanned the photon energy between 528 and 540 eV. This allowed us to record the C 1s photoline moving across the window (226 eV $< E_{kin} <$ 242 eV). By normalizing the photoline signal intensity to the pulse energy, a transmission curve was obtained. Aberration correction and absolute energy calibration were done by tuning the spectrometer to E_{kin} = 252 eV and recording C *KLL* Auger lines at 251, 253, and 254 eV presented in Fig. S1 which are independent of the photon energy and machine fluctuations. The spectrometer resolution $\approx$1.3 eV (FWHM) is a balance between resolution and electron count rate. Due to large bandwidth of the x-ray pulses $\approx$3.3 eV, the experimental resolution is not affected by the electron spectrometer resolution. In these conditions, an average of 35 electrons per XFEL pulse were detected.

Guided by the theoretical predictions, three main regions of interest were recorded at central kinetic energy of 210, 220, and 234 eV for each time delay as presented in Fig S2. In the E_{kin} = 234 eV window, the C 1s photolines from the pump and probe appear at $E_{kin} \approx$ 237 eV and

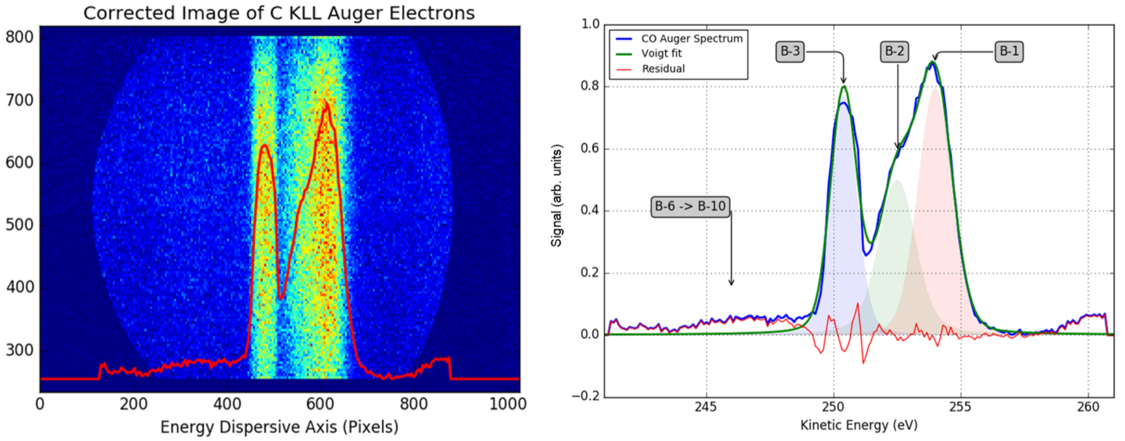

FIG. S1: Auger C KLL are used for aberration correction and absolute energy calibration of the spectrometer. (left) Aberration and transmission corrected image of the E_{kin} = 252 eV window showing the integration over 4000 images. (right) Auger spectrum extracted from the image, showing the achieved resolution of 1.3 eV.

$E_{kin} \approx 227$ eV respectively. The binding energy was calculated using the probe photon energy as reference, putting the probe C 1s photoline at ≈ 296.5 eV. The energy window centered at 220 eV, covers the high binding energy tail of the probe photoline where the core-excited state signal is anticipated. Finally, the energy window centered at 210 eV covers the Auger states.

2. *Sample delivery*

For sample delivery, an Even-Lavie pulsed valve with 30 bars backing pressure was used to deliver CO gas at the interaction point. The pulsed valve allows the delivery of high-density gas target (10^{18} molecules/m^3) at the interaction point, while keeping the pressure in the experimental chamber and spectrometer at 10^{-7} mbars. The operating conditions of the valve, i.e., backing pressure and timing of the valve, were characterized with a mass spectrometer to avoid contributions from clusters in the focus.

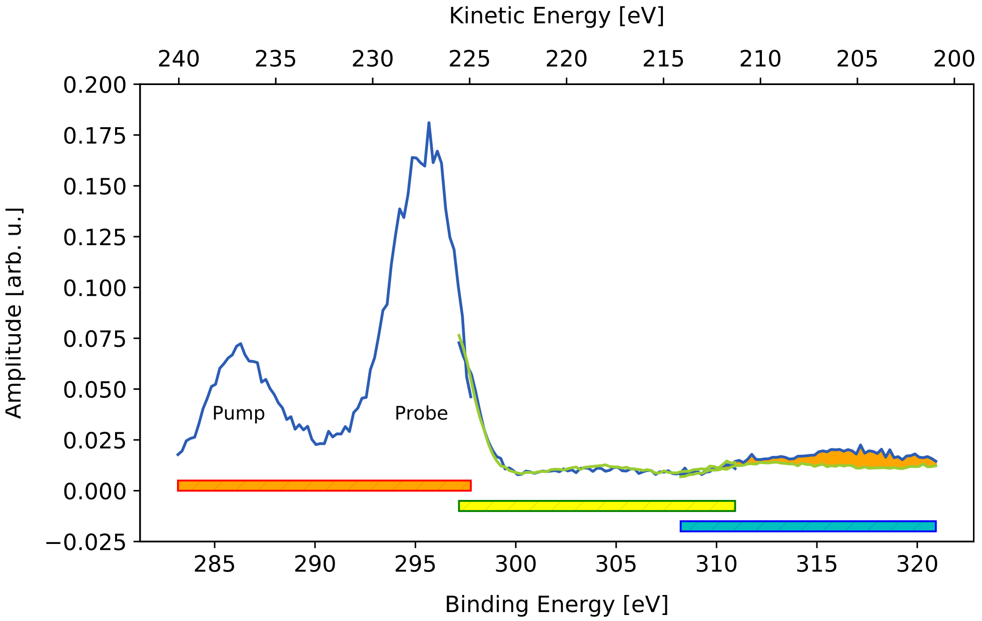

FIG. S2: Spectra measured over three energy windows with central kinetic energy at E_{kin} = 234 eV (orange), 220 eV (yellow), and 210 eV (orange). The three spectra are normalized and corrected using the XFEL parameters at each shot. Binding energy is calculated with respect to the probe photon energy at 524 eV. Orange shaded part is difference between spectra at 40 fs and -5 fs time delay.

3. Experimental conditions and pulse parameters

The LCLS XFEL offers a wealth of diagnostics tools operating at beam rate, which aides the experimenters to extract the required information for each individual event. Fig S3 presents histograms of the important measured and calculated variables required for 7 different the data sets at various time delays. The first histogram shows the total pulse intensity (pump + probe) exhibiting a distribution between 0.2 and 1 mJ. These values are measured using the Gas Monitor Detector (GMD) located between the undulators and the experiment. Although this is a very reliable measurement, it does not distinguish between the pump and probe pulse intensities. For a more detailed analysis we revert to the X-Band transverse deflecting cavity (XTCAV) which is described in detail in section S1 A 4 building on reference [1]. The analysis yields critical information required to perform time resolved XPS. Already here we present, the relative pump

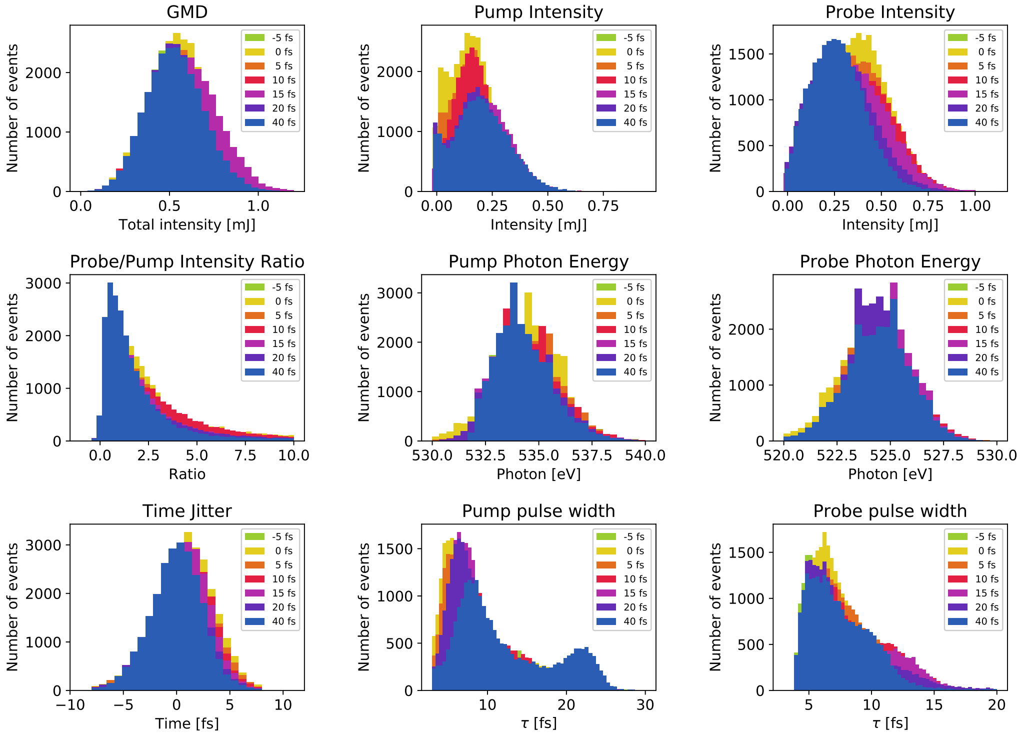

FIG. S3: XFEL parameter statistics showing total pulse intensity, photon energy, time delay fluctuation, and pulse width over 7 different time delays.

and probe pulse intensities from the XTCAV analysis. The histograms of both pulse intensities are plotted in Fig. S3. The pump shows a stable distribution across the different data sets with center around 0.2 mJ. On the other hand, the probe shows a wider distribution between 0 and 0.7 mJ, where the center of distribution changes significantly between data sets.

For XFEL sources the photon energy can be estimated from the the central electron beam energy under normal operating conditions. However, for the Fresh slice mode, the central energy of the electron beam is not a reliable value due to the chirped electron bunch, and the generation of 2 different x-ray colors. Therefore we extracted the photon energy on a shot by shot basis using linear prediction models extracted from the data itself as discussed later (S1 A 6). The resulting distribution of the pump energies is centered around the CO 1s-$2\pi^*$ resonance at 534 eV while the probe is centered around 524 eV.

As the time resolution of our experiment is of a great importance, the time delay jitter between the pulses and their widths are assessed. Using the XTCAV information, the power spectrum of the pulses were recovered (within 5 fs resolution). The time delay between the pulses is defined by two main factors, the magnetic chicane offset and the location of lasing along the electron bunch. The magnetic chicane introduces the major time difference between the pump and probe by deflecting the electron bunch in the middle of the generation process through a different path. The delay added by the chicane is very stable and exhibits fluctuations < 3 fs. The generation along the electron bunch is prone to larger time jitter as resented in S3 on the range ≈ 6 fs [2]. The pulse duration of the pump and probe pulses are also presented, showing an asymmetric distribution with a peak at ≈ 7 fs. The pump pulse distribution with pulses as long as 28 fs, which are contributed to events when lasing happened across the whole bunch, or when the pump/probe assignment have failed. Such events are discarded from the dataset.

4. *Diagnostics for timing and pulse energies*

In brief, the XTCAV is located after the undulators, where the electrons are deflected away from the lasing path. The electron bunch is dispersed along the transverse and longitudinal axis. Fig. S4 shows the image of a pristine electron bunch (left) and a spoiled one (right) that under went lasing. The dispersion along the longitudinal direction yields spectral information of the electron bunch, while dispersion along the transverse direction yields its time structure.

Both electron bunches presented are centered at 3.4 GeV with a spectral dispersion of around ≈ 40 MeV and length of ≈ 50 fs. The pump pulse is generated using the head of the bunch (10 MeV,15 fs) and the probe is at the tail (-10 MeV, -15 fs). Comparing the two XTCAV images, there is a noticeable energy spread at the expected pump and probe pulse locations. This energy spread is a direct representation of the energy loss the electrons experience when lasing. By comparing the Root Mean Square of the energy spread the two bunches at each time point, a power-vs-time profile can be extracted.

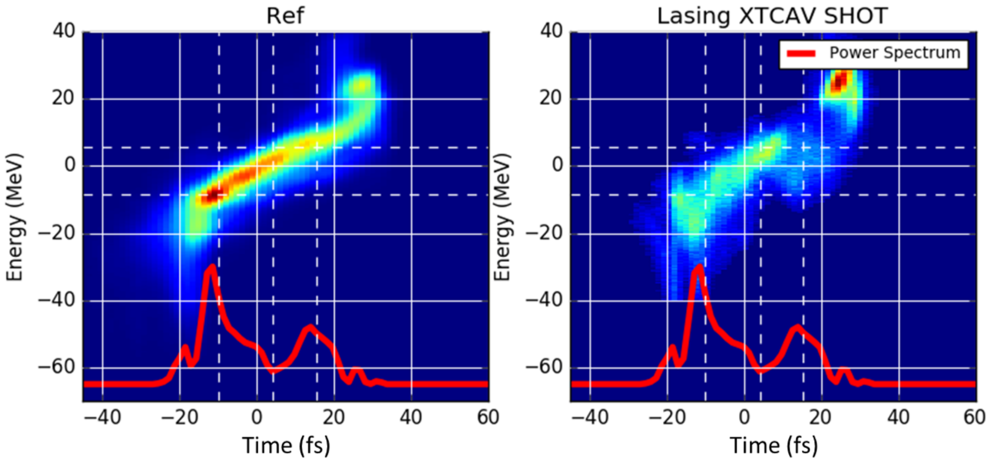

FIG. S4: XTCAV images of non-lasing pristine electron bunch (left) with 3.4 GeV central energy. Electron bunches that underwent lasing (right) exhibit energy spread at the lasing locations. The red curve shows the power spectrum in time [fs] showing the pump and probe pulses. White dashed lines guide the readers eye to the lasing positions pump (18 fs), center (+5 fs) and probe (-10 fs). Both power spectrum and dashed lines are also added to the non-lasing reference XTCAV image (left) for clarity.

$$\mathrm{P}(t) = (\sigma^2_{lasing}(t) - \sigma^2_{pristine}(t))\mathrm{I(t)}^{\frac{2}{3}} \tag{1}$$

The red curve shows the calculated power spectrum, showing two peaks at $\approx$ 18 fs and $\approx$ -10 fs representing the pump and probe pulse. Since the pulses are generated by a SASE process a simple Gaussian fit will not suffice to measure the pulses widths or estimate their time separation. We therefore use the center of mass of each of the pulses and the center of mass of the bunch. This way, the pump or probe power spectra can be isolated for pulse width and intensity assessments. The time delay between the two peaks is estimated based on the center of masses, while the pulse width is based on 1/e of the maximum intensities as follows:

$$\tau_{\text{probe}} = \mathrm{dt} \sum_{-25\text{ fs}}^{5\text{ fs}} \begin{cases} 1 & P(t) > max(P(t))/e, \\ 0 & P(t) < max(P(t))/e. \end{cases} \tag{2}$$

where τ_{probe} is the probe pulse duration, dt is increment in the time axis, and $P(t)$ is the power spectrum. τ_{probe} can be calculated using Eq. 2 with summing from 5 fs to 30 fs. For both pump

and probe, the pulse length distribution is centered around 7 fs and varies between 5 and 15 fs as shown in Fig. S3. Using the center of mass of each of the pump, the probe, and the time delay added by the chicane, the effective time delay between the two pulses was estimated. Since the time jitter is mainly due to the generation process and not changes in pathway along the magnetic chicane, the time jitter can be mainly assessed using the XTCAV data. In Fig. S3 the time jitter is presented showing a distribution spanning around 6 fs. The distribution is fairly similar for all time delays.

For reliable intensity measurements of the pump and probe pulses, the obtained power spectrum was normalized to the GMD reading in mJ. The spectrum was then integrated into two sections, the $I(\mathrm{pump})$ and $I(\mathrm{probe})$. In Fig. S3 both pulse intensities and their ratio are presented. The information extracted out of the XTCAV analysis is crucial for the normalization and data sorting to achieve the right conditions for time resolved XPS spectroscopy.

5. Ion time of flight spectrometer and photon energy calibration

In addition to the previously mentioned diagnostics, a simple ion time of flight spectrometer was installed 30 cm downstream the interaction point as independent diagnostic for the pulse intensities and photon energy. Since the spectrometer is far out of focus, the beam interaction with the gas target is fully in the linear regime. A Ne and CO gas mixture was introduced into the chamber using gas needle with a background pressure of 10^{-7} mbar. As Ne has no resonances withing the used X-ray energies used, its total ion yield would be a direct measure of the total pulse energy (pump + probe). The CO ion yield on the other hand can be resonant with the pump x-ray pulse and therefore shows a strong dependence on the x-ray pump photon energy fluctuation and flat response to the probe. For each of the runs, the CO and Ne yields are used to reference the pump photon energy relative to the O $1s - 2\pi^*$ resonance, i.e., the CO ion yield is normalized to the Ne yield and then sorted along the pump photon energy.

$$\mathrm{Int}(E_p) = \frac{\sum_i^{NShots} \frac{\mathrm{Y_{CO}}(E_p)}{\mathrm{Y_{NE}}(E_p)}}{\sum_i^{NShots}(E_p)} \tag{3}$$

Figure S5 (right) shows the CO ion yield sorted by the pump photon energy and normalized

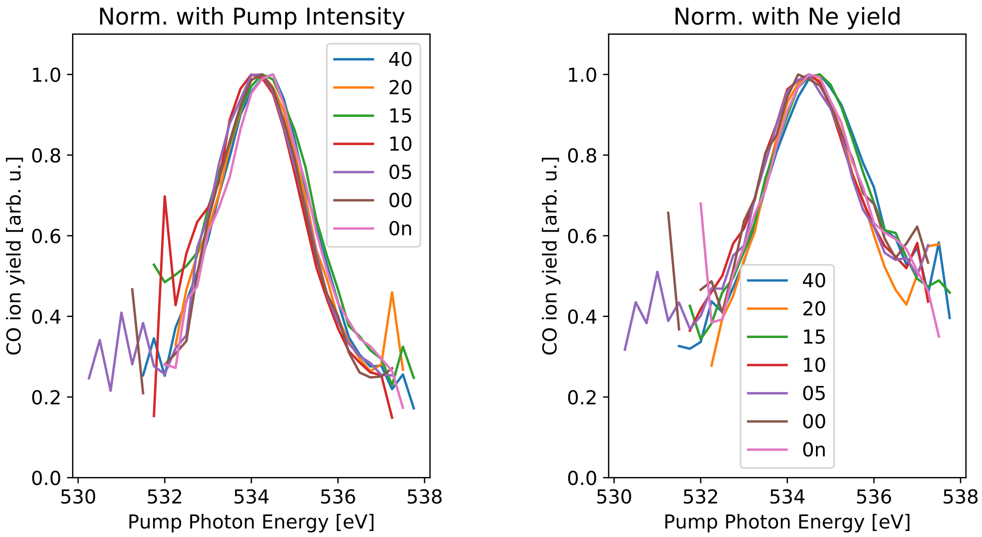

FIG. S5: CO ion yield normalized by the pump pulse intensity based on XTCAV analysis (left) and Ne ion yield (right) on a single shot basis showing a peak at 534.2 eV.

by the Ne ion yield. A clear peak can be observed at 534.2 eV, where the O 1s-2π^* resonance is anticipated.

To confirm that the pump intensities and photon energies extracted from the XTCAV and iTOF analysis is reliable, the data is sorted again using the extracted pump intensity by XTCAV as a normalization factor instead of the Ne yield. Then the curves were normalize in a similar fashion to Eq. 3. The left plot in Fig. S5 shows a slightly better contrast, which can be attributed to a more reliable normalization of the pulse and photon energy based on the XTCAV analysis. Thus we used the XTCAV information for the subsequent data analysis.

6. Data processing and analysis

The pulse generation process in the "Fresh Slice" mode is based on a SASE process and therefore it is prone to fluctuations in the pulse parameters on a shot-to-shot basis (pulse energies, photon energy, pulse width, and time delay). Further, in the "Fresh slice" mode, the adjustment of the time delay involves changes of the electron beam trajectories and machine parameters, which can yield artifacts and changes in the lasing conditions. Without the proper correction and sorting of

the data, the temporal and spectral resolution of the measured data would deteriorate significantly and make it impossible to compare the data sets at different time delays.

Therefore we start by correcting the stochastic jitter for each of the time delays using the diagnostics mentioned in the previous sections. The energy window centered at E_{kin}=234 eV is used for pump and probe photon energy estimation. A four-dimensional binning of the XFEL events is done according to the measured pump intensity, probe intensity, central photon energy (estimated from the electron beam), and time delay (XTCAV estimated time delay). For each of the bins the total XPS spectrum is extracted. We correct the XPS spectra with a Shirley background and fit two Voigt functions for the pump and probe contributions yielding the central photon energy for the individual pulses at each bin. Further, the relative signal intensity of the two photolines are compared to the estimated relative pulse intensities from the XTCAV analysis for verification.

The fits generated in the photoline window (E_{kin}=234 eV) can now be used to predict the photon energies of both the pump and probe in the other data sets based on the machine parameters and diagnostics. Considering minimal drifts in the machine within each time-delay measurements, each of the data sets could now be binned across the same four-dimensions assigning photon energy for the pump and probe to the events. To ensure that the assigned photon energies are reliable, the iTOF signals are binned based on the predicted pump photon energies where we see an increase in contrast of the signal compared to correction using only the central photon energy (as shown in Fig. S5). Both pulses were estimated to have a bandwidth $\approx$ 3.5 eV and exhibited a jitter around their nominal energy of $\pm$ 3 eV.

With the pump and probe photon energies estimated, and relative pulse intensities confirmed, the data was sorted. To ensure sufficient excitation of the sample, only pump pulses with central photon energy around the $\mathrm{O1s} - 2\pi^*$ resonance were considered (533-535 eV). As per the jitter in the probe energy, a correction in the binding energy calculation had to be considered for individual shots. Further, the individual and relative pulse energies needed to be considered in the analysis. The pump pulse energies were restricted to > 0.05 μJ and relative pump/probe intensities to < 3. If these stochastic fluctuations were not considered in the data analysis, the transient signal would washout due to off-resonant or in-sufficient excitation and the XPS spectral resolution

would deteriorate to > 7 eV.

At this point, a comparison between the different time delays could be performed. As presented in Fig. S3, each of the time delays exhibited a slightly different behavior. For example, although the total pulse energy measured with the GMD shows a fairly similar distribution between the different time delays, we notice that the distribution of the individual pulse energies show significant differences. Without the individual event diagnostics, it would be nearly impossible to compare the time dependent signals between the different data sets. Therefore, only data collected with similar parameters are considered and compared to each other. Three main parameters are considered for the comparison between the data sets, pump intensity, probe intensity, and relative pulse energy. For the temporal parameters only pulse lengths $3\,\mathrm{fs} < \tau < 10\,\mathrm{fs}$ and time delay $-3\,\mathrm{fs} < \Delta t < 3\,\mathrm{fs}$ are considered, but not sorted.

To calculate the transient signals, the data is binned over the pulse energy ratio between the pump and probe $0.5 < I(\mathrm{pump})/I(\mathrm{probe}) < 3$ with 10 bins. For each of the ratios, a corrected XPS spectrum is generated and normalized to the pulse energy and number of events in the bin. To calculate the standard error, the Bootstrapp method is used. In this iterative method, a random sample consisting of 60% (sampling and replacement method) of the total events is used to generate a new spectrum. This process is repeated 400 times. The standard error can be calculated between the residual spectra and the mean spectrum. The mean of the normalized spectra now can be compared for the various time delays in a reliable manner.

For the energy window E_{kin} = 220 eV containing the fastest dynamics from the core excited states with the smallest chemical shift, the time dependent signal is buried under the tail of the probe photo line. Here the transient signal is only expected at 0-10 fs, the spectra measured at 40 fs can be used as a reference. To calculate the transient signal and estimate error bars for the transient, a second bootstrapping estimate is performed. A random sample of each of the 400 pre-calculated spectra is used to generate a new mean spectrum at each time delay. The residual spectra obtained from subtracting the mean spectra at early time delays from the 40 fs spectrum are used as transient spectra. For each of the iterations, an integration around 298.5 eV (expected core-excited state signal) is done to estimate the signal intensity. By performing this procedure

400 times, we calculate 400 transients that can be used to estimate the error. A similar procedure is performed on the cationic states, with the main difference that the negative time delay is used as reference and two transients are calculated at 310.5 eV and 320.5 eV.

B. Theoretical method

The ultrafast electron density and nuclear dynamics in CO subsequent to O core-excitation are investigated using a two-color femtosecond X-ray pump-probe scheme. In the following we present in detail the quantum model we used to calculate the dynamics and the time-dependent chemical shift.

1. Dynamics induced by the X-ray pump pulse

The dynamics induced by the X-ray pump pulse is investigated by solving the time-dependent Schrödinger equation (TDSE) [3] :

$$\mathrm{i}\frac{\mathrm{d}\,|\Psi^{(0)}(t)\rangle}{\mathrm{d}t} = [\hat{H}_0 + \hat{V}_{\mathrm{pump}}(t)]\,|\Psi^{(0)}(t)\rangle\,, \tag{4}$$

where the upperscript (0) indicates that the X-ray probe pulse is treated at zeroth-order in time-dependent perturbation theory (TDPT). Atomic units are used in all the equations. The unperturbed Hamiltonian $\hat{H}_0$ is given by the sum of the nuclear kinetic energy $\hat{T}_{\mathrm{N}}$ and the electronic Hamiltonian $\hat{h}_{\mathrm{e}}$. The transition dipole operator in the length gauge $\hat{V}_{\mathrm{pump}}(t) = \hat{\mathbf{r}} \cdot \mathbf{E}_{\mathrm{pump}}(t)$ describes the interaction of one core electron in the molecule with the pump pulse. The latter is a Gaussian pulse defined as

$$\mathbf{E}_{\mathrm{pump}}(t) = E_0 \sin\left(\omega_0^{\mathrm{pu}}(t - t_0)\right)\exp\left[-\frac{4\log 2(t-t_0)^2}{t_{\mathrm{FWHM}}^2}\right]\boldsymbol{\epsilon}, \tag{5}$$

where E_0 is the amplitude, t_0 the temporal center, ω_0^{pu} the central frequency, t_{FWHM} the full width at half maximum and $\boldsymbol{\epsilon}$ the polarization of the pulse which is considered to be perpendicular to the molecular axis, corresponding to the direction of maximum excitation. Non-adiabatic couplings are not included in our model. The molecular wavefunction at zeroth-order in perturbation

theory is expressed as:

$$|\Psi^{(0)}(t)\rangle = |\chi_{\rm gs}(t)\rangle \otimes |{\rm gs}\rangle + |\chi_{\rm ce}(t)\rangle \otimes |{\rm ce}\rangle + \sum_J \sum_a \int {\rm d}\varepsilon_a \, |\chi_{\varepsilon_a J;a}(t)\rangle \otimes |\varepsilon_a J; a\rangle \,. \quad (6)$$

In Eq. 6 $|\chi_{\rm gs}(t)\rangle$, $|\chi_{\rm ce}(t)\rangle$ and $|\chi_{\varepsilon_a J;a}(t)\rangle$ are the nuclear wavepackets in the ground, the core-excited and the Auger states, respectively, while $|{\rm gs}\rangle$, $|{\rm ce}\rangle$ and $|\varepsilon_a J; a\rangle$ are the corresponding electronic states. The core-excited state is charaterized with a hole in the 1s orbital of the O atom. The Auger state resulting from the decay of the core-excited state is given by a cationic state with one hole in the valence shell a and is associated with a continuum state of energy ε_a and angular momentum J.

The equations of motion for the nuclei for this ansatz are:

$$\begin{aligned} {\rm i}\frac{{\rm d}\,|\chi_{\rm gs}(t)\rangle}{{\rm d}t} &= [\hat{T}_{\rm N} + E_{\rm gs}(\mathbf{R})]\,|\chi_{\rm gs}(t)\rangle + \sum_i \langle {\rm gs}|\hat{V}_{\rm pump}(t)|{\rm ce}; i\rangle\,|\chi_{\rm ce;i}(t)\rangle \\ {\rm i}\frac{{\rm d}\,|\chi_{\rm ce}(t)\rangle}{{\rm d}t} &= \left[\hat{T}_{\rm N} + E_{\rm ce}(\mathbf{R}) - {\rm i}\frac{\hat{\Gamma}_{\rm ce}}{2}\right]|\chi_{\rm ce}(t)\rangle + \langle {\rm ce}|\hat{V}_{\rm pump}(t)|{\rm gs}\rangle\,|\chi_{\rm gs}(t)\rangle \\ {\rm i}\frac{{\rm d}\,|\chi_{\varepsilon_a J;a}(t)\rangle}{{\rm d}t} &= [\hat{T}_{\rm N} + E_{\varepsilon_a;a}(\mathbf{R})]\,|\chi_{\varepsilon_a J;a}(t)\rangle + \langle \varepsilon_a J; a|\hat{h}_{\rm e}|{\rm ce}\rangle\,|\chi_{\rm ce}(t)\rangle \,. \end{aligned} \quad (7)$$

In Eq. 7, $E_{\rm gs}(\mathbf{R})$, $E_{\rm ce}(\mathbf{R})$ and $E_{\varepsilon_a;a}(\mathbf{R})$ are respectively the potential energy curves of the ground, the core-excited and the Auger states, the latter being independent on the angular momentum of the Auger electron. The Auger decay is represented phenomenologically via an Auger decay width $\hat{\Gamma}_{\rm ce}$. In order to interrogate the dynamics of the left parent ion after the Auger decay, we are interested in the probability for the molecule to be in a particular Auger state. We use the Auger decay rates for each channel $\gamma_{\varepsilon_a;a}$ from the measurements of Ref. [4], and it is convenient to define a weighted amplitude

$$|\chi_{\varepsilon_a;a}(t)\rangle = \sum_J \frac{\langle {\rm ce}|\hat{h}_{\rm e}|\varepsilon_a J; a\rangle}{\sqrt{\gamma_{\varepsilon_a;a}}}\,|\chi_{\varepsilon_a J;a}(t)\rangle \,, \quad (8)$$

to describe the dynamics after the Auger decay, whose weights are given by the corresponding Auger transitions. The weighted amplitude can be evolved with the following equation of motion, assuming that $\langle {\rm ce}|\hat{h}_{\rm e}|\varepsilon_a J; a\rangle$ is not changing significantly with the internuclear distance around the equilibrium geometry:

$${\rm i}\frac{{\rm d}\,|\chi_{\varepsilon_a;a}(t)\rangle}{{\rm d}t} = [\hat{T}_{\rm N} + E_{\varepsilon_a;a}(\mathbf{R})]\,|\chi_{\varepsilon_a;a}(t)\rangle + \sqrt{\gamma_{\varepsilon_a;a}}\,|\chi_{\rm ce}(t)\rangle \,, \quad (9)$$

in which the partial Auger decay widths are written as

$$\gamma_{\varepsilon_a;a} = \sum_J | \langle \mathrm{ce} | \hat{h}_\mathrm{e} | \varepsilon_a J; a \rangle |^2, \tag{10}$$

and the total Auger decay width as

$$\hat{\Gamma}_\mathrm{ce} = 2\pi \sum_a \int \mathrm{d}\varepsilon_a \gamma_{\varepsilon_a;a} \delta(E_{\varepsilon_a;a} - E_\mathrm{ce}). \tag{11}$$

In Eqs. (10) and (11), we do an incoherent sum over the angular momentum. We neglect the phase of the states in Eq. (10) as the Auger states are separated in energy (see Table S1) and thus do not lead to interference during the propagation. In the experiment, we are not measuring the angular momentum distribution such that this approximation is justified in our model.

2. *Photoelectron yield at first order of time-dependent perturbation theory*

The coupled nuclear and electron density dynamics are probed with a second time-delayed femtosecond X-ray pulse, treated at first order in TDPT. The TDSE thus reads

$$\mathrm{i}\frac{\mathrm{d}\,|\Psi(t)\rangle}{\mathrm{d}t} = [\hat{H}(t) + \hat{V}_\mathrm{probe}(t)]\,|\Psi(t)\rangle\,, \tag{12}$$

with $\hat{H}(t) = \hat{H}_0 + \hat{V}_\mathrm{pump}(t)$. The first-order correction to the ansatz for the molecular wavefunction Eq. (6) involves the amplitude of the core-ionized states created by the X-ray probe pulse:

$$\begin{aligned} |\Psi^{(1)}(t)\rangle = &\sum_s \int \mathrm{d}\varepsilon\, |\chi_{\varepsilon;s[\mathrm{gs}]}(t)\rangle \otimes |\varepsilon; s[\mathrm{gs}]\rangle + \sum_s \int \mathrm{d}\varepsilon\, |\chi_{s[\mathrm{ce}]}(t)\rangle \otimes |\varepsilon; s[\mathrm{ce}]\rangle \\ &+ \sum_s \sum_a \int \mathrm{d}\varepsilon \int \mathrm{d}\varepsilon_a\, |\chi_{s[\varepsilon_a;a]}(t)\rangle \otimes |\varepsilon; s[\varepsilon_a; a]\rangle\,. \end{aligned} \tag{13}$$

The index s labels the main core-hole state as well as its satellites, for which the main state after core ionization is accompanied by a valence-to-valence excitation. The symbol $s[i]$ stands for the satellite state(s) created through core-ionizing the initial state i, which can be either the ground, the core-excited or an Auger state. The first term in Eq. (13) denotes the states created by ionizing the C1s core electron in the ground state together with the emitted photoelectron of energy ε. The second term corresponds to double core-hole states, populated through the C-1s-electron ionization of the core-excited state already created by the pump pulse. The third term denotes the

core-hole states generated by ionizing in the core the Auger states at the C site, defined by the average states given in Eq. (8).

The photoelectron yield at a particular photoelectron energy ε is obtained by solving the equation of motion for the final core-ionized states populated by the X-ray probe pulse within the rotating wave approximation and the short pulse approximation, and it is proportional to

$$P_{\varepsilon,i} = \sum_R P_{\varepsilon,i}(R) \propto \tau^2_{\text{probe}} \mid \langle \varepsilon; s[i]|\Omega_0|i\rangle \mid^2 \sum_R |\chi_i(R, t_0)|^2, \tag{14}$$

where $s[i]$ denotes the core-hole state originating from either the ground, the core-excited or an Auger state, and R the internuclear distance. Ω_0 and τ_{probe} are the amplitude and the pulse length of the probe pulse centered at t_0.

In the present case, coherences do not play a significant role and we treat all the different ionization channels coming from the different Auger paths independently, as they are quite separated in energy, see table S1. Hence, Auger electrons from different channels will be emitted to the continuum with different energies due to energy conservation, with a broadening given by the Auger decay lifetime (0.158 eV), having no overlap among them. This explains why we can separate the ionization processes in Eq. (14). We perform an incoherent sum over the photoelectron spectra associated with different nuclear geometries (i.e. different internuclear distance R). Moreover, we consider that the probe pulse suddenly removes the core electron, such that the dipole matrix elements $\langle \varepsilon; s[i]|\Omega_0|i\rangle$ can be split in two factors: $\langle \varepsilon|\Omega_0|1s\rangle$ that involves the dipole coupling between the 1s orbital and the photoelectron, and $\langle \Phi_{s[i]}|\Phi_i^{N-1}\rangle$ which corresponds to the overlap between electronic wavefunctions at $N-1$ electrons, where N is the number of electrons in the neutral molecule. In particular, $\Phi_{s[i]}$ and Φ_i^{N-1} are the electronic wavefunctions of the final core hole states and the initial state, which again can be either the ground, the core-excited or an Auger state. The description of the continuum state and its interaction with the molecular cationic state is a very challenging task. In the case of highly energetic photoelectrons (here the photon energy is far above the ionization thresholds, and the photoelectron energy is around 224 eV), the cross section is almost independent on the energy and the matrix element $\langle \varepsilon|\Omega_0|1s\rangle$ is almost constant. Therefore, in this high photon energy regime, the term that is mainly responsible for the modulations in

the XPS spectra is $\langle \Phi_{s[i]} | \Phi_i^{N-1} \rangle$. We evaluate this overlap within the sudden approximation, i.e. Φ_i^{N-1} is obtained by suddenly removing a 1s electron (the other orbitals being frozen), while is $\Phi_{s[i]}$ the true electronic wavefunctions, i.e. for which the orbitals have been relaxed in the presence of the core vacancy. The final XPS spectrum is obtained by doing a convolution with a Gaussian function with full width at half maximum of 3.5 eV to take into account the probe pulse bandwidth. The population in the Auger states depends on the Auger electron energy ε_{a}, such that for these states the photoelectron yield is obtained by integrating Eq. (14) over ε_{a}.

3. *CASSCF and CI potential energy curves*

In this section, we detail the calculation of the potential energy curves (PECs) of the ground, the core-excited, the Auger states as well as those states resulting from the core ionization at the C site. The electronic configuration of the neutral ground state of CO is:

$$(1\sigma)^2(2\sigma)^2(3\sigma)^2(4\sigma^*)^2(5\sigma)^2(1\pi)^4(2\pi^*)^0(6\sigma^*)^0, \tag{15}$$

where the 1σ and 2σ orbitals correspond to the O1s and C1s atomic orbitals, respectively.

In order to describe accurately the electron correlation that play an important role especially in core-hole states, we calculate the lower energetic states at the complete active space self-consistent field (CASSCF) level of theory, while a configuration interaction (CI) calculation is performed to reach the highly excited states.

The PECs of the lower energetic states (< 20) are first calculated at the CASSCF level of theory for each symmetry (Φ, Π, Δ and $\Sigma^{\pm}$) and spin configuration separately. In particular, the doublet core-hole states originating from either the ground or the core-excited singlet states, as well as the singlet and triplet core-hole states originating from the doublet Auger states are considered. The calculations are performed in $\mathrm{C_{2v}}$ symmetry using the correlation-consistent polarized valence quadruple-zeta (cc-pVQZ) basis set of Dunning using the Molpro quantum chemistry package [5]. The active space comprises all core and valence orbitals given in Eq. (15). A single core-excited state is calculated, while a state-average CASSCF calculation is performed for the Auger and core-ionized states because of the presence of many states closed in energy. The electronic

configuration as well as the calculated vertical binding energy of the core-excited and Auger states at equilibrium geometry of the ground state are given in Table S1, and they are compared to values from the experimental paper [4] that we use as a reference to determine the Auger states that mostly contribute to the dynamics. For core-hole states, the 1s orbital is frozen and its occupancy is fixed to 1. For each geometry, the molecular orbitals (MOs) of the core-hole states are diabatized with respect to the MOs of the state from which they originate – through core ionization by the probe pulse – in order to maximize their overlap.

According to Table S1, the Auger states span a large energy window of about 70 eV (between the lowest and the highest Auger state), and the satellite states may also be far in energy compared to the main core-hole state. A state-average CASSCF calculation is restricted to a reasonable number of states. In order to describe the highly excited states, we thus use the MOs optimized at the CASSCF level to perform a CI calculation in C_1 symmetry using the OpenMolcas quantum chemistry package [7]. The double-core-hole states, produced by core-ionization of the core-excited state by the probe pulse, are calculated using the generalized active space self-consistent field (GASSCF) method, keeping frozen the starting MOs that have been previously optimized at the CASSCF level with Molpro. Again we calculate the PECs for each symmetry and each spin state separately. We calculate 200 PECs for each spin and symmetry core-hole state.

4. *Role of satellite states in the photoelectron yield*

The energy calculations of core-hole states depend on the strong electron relaxation and correlation effects in the presence of a core vacancy. The formation of a bunch of satellite states while a core hole is created is the rule more than the exception. Since satellite states are close in energy to the main core-hole state, they may play a relevant contribution to the XPS spectrum.

The strong mixing between satellite states makes it impossible to associate one particular core-hole state to the state from which it originates. For example, core-ionization of an Auger state V^{-1} (with one hole in the valence shell V) does not only produce a core-hole state of electronic configuration $1\mathrm{s}^{-1}\mathrm{V}^{-1}$ but also core-hole states with other valence electronic configurations. To

State	Configuration	Vertical binding energy [eV] Expt.	Present	Decay width [meV]
		Core-excited state		
CO^* ($^1\Pi$)	$O1s^{-1}2\pi^*$	534.2 [6]	536.55	158 [6]
		Cationic Auger state		
CO^+ ($^2\Phi$)	$1\pi^{-2}2\pi^1$	30.57	29.15	4.502
CO^+ ($^2\Pi$)	$1\pi^{-1}$	17.74	16.53	2.233
	$5\sigma^{-2}2\pi^1$	24.04	22.91	0.298
	$4\sigma^{-1}5\sigma^{-1}2\pi^1$	28.77	27.40	0.536
	$1\pi^{-2}2\pi^1$	29.42	28.02	1.482
	$4\sigma^{-1}5\sigma^{-1}2\pi^1$	29.51	28.71	0.868
	$1\pi^{-2}2\pi^1$	31.87	30.52	3.527
	$4\sigma^{-2}2\pi^1$	39.87	38.87	1.247
	$3\sigma^{-1}4\sigma^{-1}2\pi^1$	59.50	59.77	0.548
	$3\sigma^{-1}4\sigma^{-1}2\pi^1$	62.08	61.03	0.877
	$3\sigma^{-2}2\pi^1$	83.70	80.49	0.378
CO^+ ($^2\Delta$)	$5\sigma^{-1}1\pi^{-1}2\pi^1$	24.20	25.42	0.400
	$4\sigma^{-1}1\pi^{-1}2\pi^1$	34.52	32.98	2.627
	$3\sigma^{-1}1\pi^{-1}2\pi^1$	50.93	48.88	0.310
	$3\sigma^{-1}1\pi^{-1}2\pi^1$	58.92	57.16	1.604
CO^+ ($^2\Sigma^+$)	$5\sigma^{-1}$	14.40	13.06	0.157
	$4\sigma^{-1}$	20.45	19.17	0.269
	$4\sigma^{-1}1\pi^{-1}2\pi^1$	34.23	32.37	1.284
	$3\sigma^{-1}$	43.12	40.96	0.076
	$3\sigma^{-1}1\pi^{-1}2\pi^1$	55.64	55.43	0.349
CO^+ ($^2\Sigma^-$)	$4\sigma^{-1}1\pi^{-1}2\pi^1$	35.20	32.98	1.574

TABLE S1: The energy of the ground state is taken as the reference for the vertical binding energies, which is calculated at the equilibrium internuclear distance ($R_{\mathrm{eq}} = 1.12$ Å) of the ground electronic state. Most of the partial decay widths are taken from [4] and are normalized such that the total Auger decay width of the core-excited state is 158 meV. Note that the partial decay widths in Ref. [4] are obtained by measuring the Auger electron energy, while no information about the angular momentum distribution is provided in this experiment.

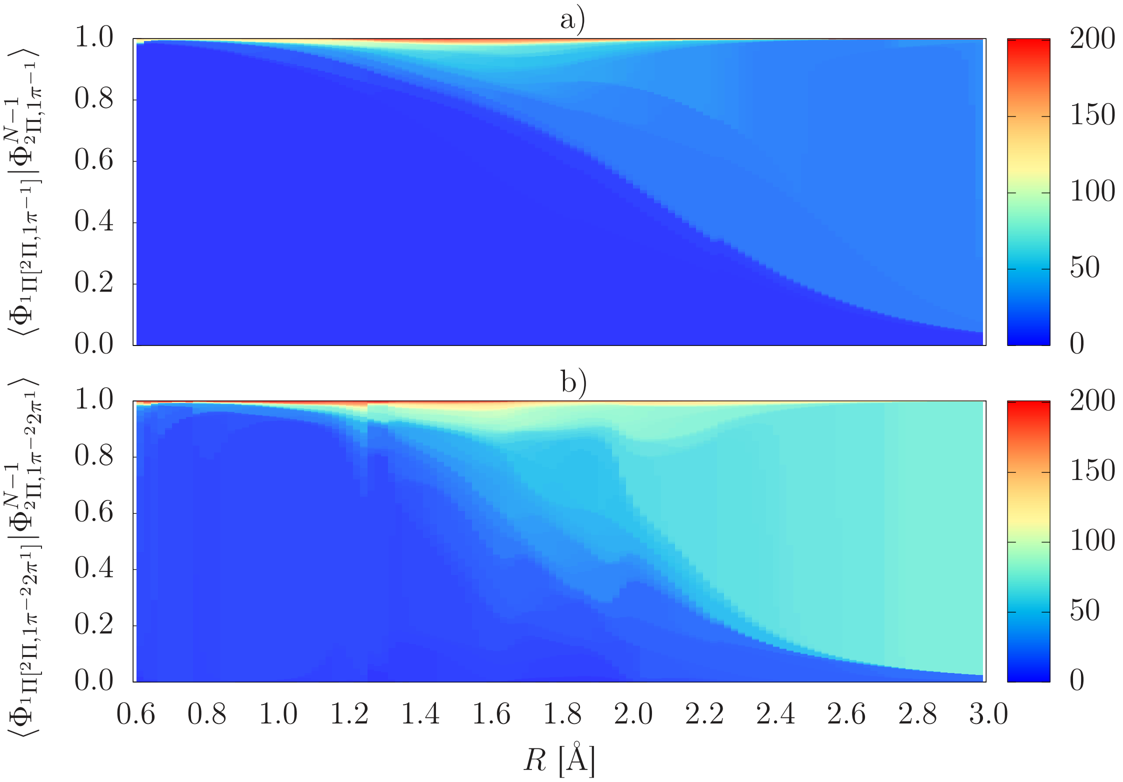

FIG. S6: Overlap between the singlet core-hole state of Π symmetry with the singlet core-hole state obtained by removing the C 1s electron within the sudden approximation from a) the Auger state $1\pi^{-1}$, and b) the doublet Auger state $1\pi^{-2}2\pi^1$.

illustrate the importance of satellite states to the XPS spectrum, Fig. S6 shows the overlap between the core-hole states of Π symmetry, including the electron relaxation in the presence of a core vacancy, with the singlet core-hole state obtained within the sudden approximation, i.e removing the C 1s electron from the 1st Π Auger state (Panel a) and from the 7th Π Auger state (Panel b) as a function of the geometry. For short internuclear distances ($R < 1.2$ Å) the low-lying core-hole states dominate. However, as the bond stretches the contribution of highly excited satellite states increases, especially in the case where the initial (Auger) state is itself high in energy (Panel b). The same tendency is observed for the other symmetry and spin states but are not shown here. This result demonstrates that satellite states are essential to provide a realistic description of core ionization processes while the molecule dissociates.

5. *Numerical implementation*

The equations of motion for the nuclei (7) and (9) are implemented using the fourth-order Runge-Kutta algorithm. The initial wavepacket is the ground vibrational state of the ground electronic state. These equations are solved for each energy of the Auger electron energy. A multi-grid in energy is used, and the number of points is defined as $NE_{\rm a} = (100 \times \Gamma_{\rm ce})/dE_{\rm a}$, with $dE_{\rm a} = 0.02$ eV the energy resolution. A grid in energy is defined for each Auger state and is centered at the kinetic energy of the Auger electron at the equilibrium internuclear distance of the ground state ($R_{\rm eq} = 1.12$ Å). The total Auger decay width and the partial Auger decay widths are taken from previous experimental studies [4, 6]. The PECs calculated between $R = 0.6$ Å and 3.0 Å are extrapolated up to 13.2 Å with a grid step of $dR = 4.2 \times 10^{-3}$ Å. The nuclear propagation is performed up to 100 fs using a time step of $dt = 2.4 \times 10^{-4}$ fs. The pump pulse is centered at $t_0 = 50$ fs and its central frequency $\omega_0^{\rm pu} = 536.55$ eV is set to the resonance $\rm gs \rightarrow CO(1s^{-1})2\pi^*$. The pulse of length $t_{\rm FWHM} = 10$ fs (energy bandwidth of 0.182 eV) and intensity $I = 10^{16}$ $\rm W/cm^2$ is polarized perpendicular to the molecular axis. The transition dipole moment between the ground and the core-excited state amounts to 0.1518 Debye at the CASSCF level at $R_{\rm eq} = 1.12$ Å. Because the initial nuclear wavepacket in the ground electronic is localized, we assume the transition dipole moment to be independent of the internuclear distance R. The time-resolved XPS spectra associated with ionization of the ground, core-excited, and Auger states are calculated, see Eq. (14), from the overlap between the relaxed core-hole states and the ones obtained within the sudden approximation and the dynamical nuclear wavepackets.

S2. SUPPLEMENTARY DISCUSSION

A. X-ray induced electron redistribution

In order to provide a deeper insight into the x-ray induced ultrafast electron dynamics, we quantify changes in the electron density at the early times after core-excitation as well as after Auger decay of the transient state. In particular, an analysis of the Mulliken charges $Q_{\rm m}$ allows us

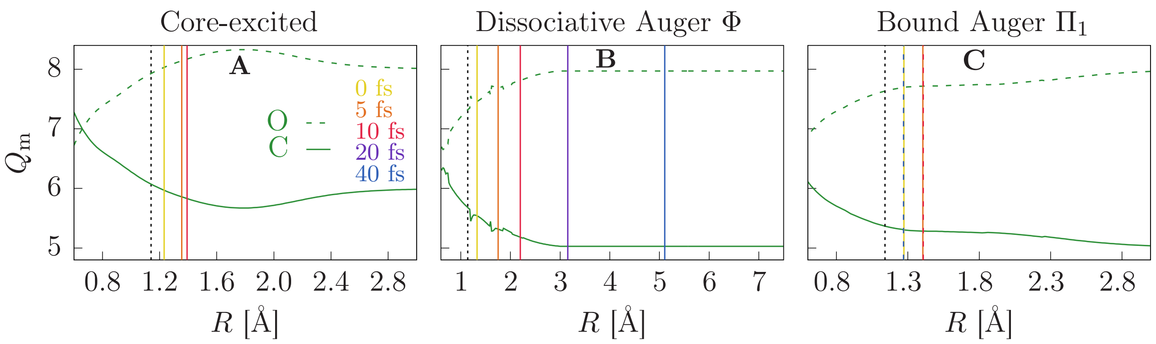

FIG. S7: Mulliken charges $Q_{\rm m}$ in the C (solid line) and O (dashed line) in the (A) core-excited, (B) dissociative Φ and (C) lowest bound Π states. The average position – the expectation value of the internuclear distance $\langle R\rangle$– of the nuclear wavepacket is indicated for different characteristic times (colored vertical bars). The vertical dotted black line correspond to the equilibrium distance of the ground electronic state and is displayed to guide the eye.

to get a deep insight into electron screening effects in the presence of a core hole and to localize the hole in the valence shells of the Auger states. These partial electronic charges are calculated for each internuclear distance with the OpenMolcas quantum chemistry package [7]. The difference between the Mulliken charges in these states and the ones at the equilibrium geometry in the ground state ($R\sim 1.14$ Å), denoted $\Delta Q_{\rm m}$, are displayed in panels S7A, S7B and S7C. Therefore, a positive (negative) $\Delta Q_{\rm m}$ corresponds to an increase (decrease) in the electron density in the state under consideration compared to the ground state. The vertical bars in S7 indicate the average position of the nuclear wavepacket (i.e. the expectation value of the internuclear distance $\langle R\rangle$) for some characteristic times. In this way, we can relate this observable calculated for each internuclear distance R to the time domain.

In the ground state the $Q_{\rm m}$ amount to 5.95 and 8.05 at the C and O sites, respectively, while at the instant of excitation, the charges in the core-excited state amount to 5.97 and 8.03 at the C and O sites, see Fig. S7A. The density thus increases at the C site compared to the ground state due to the excitation of the O1s electron to a delocalized $2\pi^*$ orbital. After 5 fs $Q_{\rm m}$ amount to 5.86 and 8.14 at the C and O sites, respectively. The bond stretches even more such that after 10 fs the partial charges at the C and O atoms amount to 5.83 and 8.17. This results from a flow

of electron density along the chemical bond towards to O, leading to a complete screening of the core hole. This decrease in electron density at the C site leads to an increase of the binding energy of the C1s electron and thus to a chemical shift towards higher energy in the time-resolved XPS spectrum. This theoretical analysis allows us to detect subtle ultrafast changes in the electron density in the transient core-excited state concomitant to Auger decay, and allows us to interpret the experimental time-dependent chemical shifts.

Auger decay of the core-excited state leads to the filling of the core vacancy and to the ejection of an Auger electron, such that the hole ends up in the valence shells. Using the Mulliken charges we are able to follow in real-time the localization of the hole in the Auger states. For the sake of clarity we focus our analysis on the dominant dissociative (Φ) and bound (Π_1) Auger states, see Fig. S7B and Fig. S7C, respectively. In both cationic states the charges at both the C and O sites are smaller compared to the ones in the ground state.

In the course of molecular fragmentation in the dominant dissociative Φ Auger state, the partial charges at the O (C) site increase (decrease) up to 20 fs and then remain constant: Right after Auger decay Q_{m} amount to 5.54 and 7.46 at the C and O sites, respectively, and reach 5.03 and 7.97 after 20 fs. These results demonstrate that the valence hole is shared between the two atoms in the early times of the dynamics, and it is trapped at the C site after molecular fragmentation. This reduces the screening of the nuclear charge of the C atom, which in turn increases the binding energy of the core electron.

On the other hand, we expect a totally different behavior for bound and quasi-bound Auger states, which are the lowest states of Π and Δ symmetries and the two lowest Σ^+ states [4]. Several vibrational states are populated in those channels presenting small variations in the bond distance. By performing the Mulliken population analysis in the dominant bound Auger state (Π_1), see Fig. S7C, we observe that molecular vibration produces slight oscillations in the partial charge around 5.30 and 7.70 at the C and O sites, respectively. This shows that the valence hole remains delocalized on both sites during the nuclear dynamics, such that no significant chemical shift in the time-resolved XPS spectra of states is expected.

Therefore, this thorough analysis of the electron density at each atomic sites allows us to get

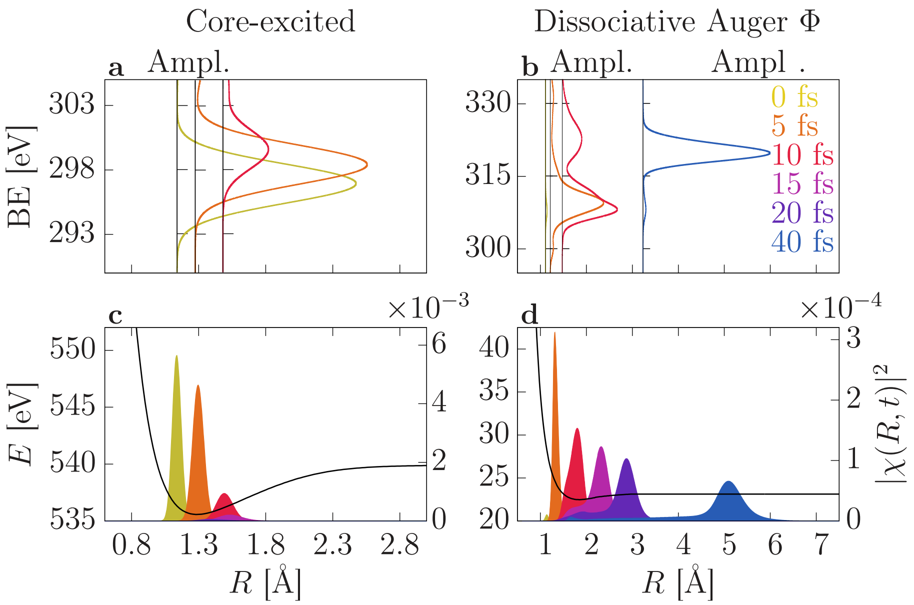

FIG. S8: Impact of the pulse duration on the nuclear wavepacket dynamics and on the time-resolved chemical shifts. The time-resolved XPS spectra for 1 fs excitation pulse of the (a) core-excited state, and (b) dissociative Φ state are displayed for different time delays. Panels (c) and (d) show the potential energy curves with the nuclear wavepacket at different time delays in the core-excited and the Φ Auger states, respectively.

a movie of electron rearrangements right after core-excitation, during and after the Auger decay, and to shed light on the ultrafast chemical shifts in the time-resolved XPS spectra.

B. Nuclear wavepacket distribution

Here we discuss the effect of the excitation pulse length on the shape of the nuclear wavepacket in the core-excited state and the nuclear wavepacket shape and propagation on the dynamical chemical shifts. We note that the core-excited state width is 0.158 eV (4.17 fs lifetime) and the energy vibrational splitting is approximately 0.115 eV with a period of $\sim$ 36 fs. Therefore, the excitation with a 1-fs FWHM pulse (1.8 eV bandwidth) may excite many vibrational states, in contrast with the 10-fs FWHM pulse (0.18 eV bandwidth).

Upon electronic excitation of the oxygen site with the pump pulse, a chemical shift at the C-site of 2.4 eV is calculated with respect to the ground state. This chemical shift is attributed to electron

screening of the core vacancy created by the excitation pulse. Already with the limited experimental spectral resolution, the chemical shift from the core-hole state was observed in the data with a similar spectral and temporal behavior as the theoretical predictions. In the calculated spectra, an additional time-dependent shift of ~ 1.1 eV was predicted. The dynamics and presence of this time-dependent shift can be attributed to the bimodal shape of the nuclear wavepacket launched in the excited state potential energy surface (PES). The difference, meaning the absence of the time-dependent shift, between theory and experiment can be justified by the different boundary conditions. In the experiment we use SASE pulses which prevent us from creating a well-defined wavepacket and thus wipe out the additional time-dependent chemical shifts.

Let us further discuss the relationship between the wavepacket shape and dynamical chemical shift of ~ 1.1 eV. In our model the core excitation is induced by a transform-limited pulse of 10 fs full-width at half maximum (FWHM), leading to the formation of a well-defined bimodal nuclear wavepacket distribution in the transient state, see Fig. 3d. Further, the calculations show that the dynamical shift correlates directly with the center of mass motion of the wavepacket. The bimodal nuclear wavepacket additionally leaves its imprint on the dynamics in the cationic states populated through Auger decay: The nuclear wavepacket in the dissociative Φ Auger state does not exhibit a Gaussian shape but is also structured from the early time of the dynamics, see Fig. 3e. For comaprison, using a shorter pulse of 1 fs FWHM duration, the nuclear wavepacket in the core-excited state is more localized in space and it conserves its Gaussian shape, see Fig. S8c, as interferences in the core-excited state are now less pronounced. The ultrafast chemical shift arises from the purely electronic excitation (i.e. at 0 fs) and amounts to 1.85 eV. Over time, an additional ultrafast chemical shift of 1.51 eV develops in the core-excited state due to the bond stretching in the presence of a core-hole, as shown in Fig. S8a.

The dynamics and the time-resolved XPS spectra of the dissociative Φ Auger state are displayed in Panels S8d and S8b, respectively. A double peak structure appears in the time-resolved XPS signal while the molecule dissociates: At 5 fs, the main spectral feature is at 309.46 eV, while for longer time delays another peak raises at higher binding energy at ~ 319.75 eV. Again, the lower and higher peaks can be explained from the nature of the Auger states, being either bound

or dissociative, respectively.

S3. SUPPLEMENTARY REFERENCES

---

[1] C. Behrens, F.J. Decker, Y. Ding, V.A. Dolgashev, J. Frisch et al., "Few-femtosecond time-resolved measurements of X-ray free-electron lasers," Nat. Comm. **5**, 3762 (2014)

[2] Z. Guo, M. W. Guetg, Y. Ding, A. Marinelli, J. Wu et al., "Simulation analysis and optimization of fresh-slice multistage free-electron lasers," Phys. Rev. Accel. Beams **23**, 031304 (2020)

[3] A. Picón, "Time-dependent Schrödinger equation for molecular core-hole dynamics," Phys. Rev. A **95**, 023401 (2017)

[4] M. N. Piancastelli, M. Neeb, A. Kivimäki, B. Kempgens, H. M. Köppe et al., "Vibrationally resolved 1s $\rightarrow 2\pi$ decay spectra of CO at the C and O K-edges: experiment and theory," J. Phys. B: At. Mol. Opt. Phys. **30**, 5677 (1997)

[5] H.-J. Werner, P. J. Knowles, G. Knizia, F. R. Manby and M. Schütz, "Molpro: a general-purpose quantum chemistry program package," WIREs Comput. Mol. Sci. **2**, 242 (2012)

[6] M. Coreno, M. de Simone, K. C. Prince, R. Richter, M. Vondracek et al., "Vibrationally resolved oxygen $K \rightarrow \Pi^*$ spectra of O_2 and CO," Chem. Phys. Lett. **306**, 269 (1999)

[7] I. F. Galván, M. Vacher, A. Alavi, C. Angeli, F. Aquilante et al., OpenMolcas: From Source Code to Insight. J. Chem. Theory Comput. **15**, 5925–5964 (2019)
